# On the Prevailing Taste for Hypotheses in Medicine, with a Review of Ancient Practice in Acute Diseases

**Published:** 1800-08

**Authors:** S. C. Blyth

**Affiliations:** Army Hospital, Yarmouth


### *To the Editors of the Medical and Phyſical Journal.*

GENTLEMEN,

THE early and polite attention which you did me the honour to pay a former Eſſay, emboldens me to ſubmit the following remarks to your conſideration. I am,

GENTLEMEN,

With great reſpect,

Your very humble Servant,

S. C. BLYTH.

*Army Hoſpital, Yarmouth,*
*July* 14, 1800.

### *On the prevailing Taste for Hypotheses in Medicine, with a Review of ancient Practice in acute Diseases.*

IT is fomewhat ftrange, that though in many arts and fciences, improvement has advanced in a ftep of regular progreffion from the firft moment of their invention, in others it has kept no pace with time, and we look back to ancient excellence with wonder, not unmixed with awe.

Medicine feems to be one of thofe ill-fated arts, whofe improvement bears no proportion to its antiquity; for it is lamentably true, that although Anatomy has been better illuftrated, the Materia Medica enlarged, and Chemiftry almoft created fince the revival of letters, we are very little before the ancients in the penetration which difcovers the precife nature of a diforder, or in fkill to apply the remedy.

Perhaps it may be on account of thofe very advantages, that modern practitioners are inferior to thofe of ancient times. We have fo many collateral helps, that we feldom ftudy Nature in her original character, but fee her at fecond hand, often deformed by the injudicious narrator, or difguifed and fafhioned by the plaftic hand of the Theorift, to fuit a favourite hypothefis.

Hippocrates, the father of his art, and the prince of phyficians, has faid, that a phyfician fhould be the minifter of Nature; and is it not our departing from Nature, in compliment to new theories, that modern practice appears fo diminutive, compared to that of the illuftrious native of Cos? A review of his mode of treating acute diforders may juftify thefe remarks.

The celebrated Grecian's firft principle was, "That the end of phyfic is either to carry off difeafes, or to moderate their violence."—Accordingly, his indications in acute difeafes were "to mitigate the fymptoms, when too violent, and to affift Nature in her bufinefs of concoction or evacuation of the febrile matter."—Having taken thefe indications from Nature, when the fever was irregular, and had no certain type, he waited without doing any thing till it became regular, and then he proceeded according to the predominancy of the fymptoms.

Confidering a fever in every circumftance as an effort of Nature to rid herfelf of a load, he never attempted *to cure it*,—that is, he never wifhed to arreft the febrile commotion altogether.—He reftrained thefe efforts by bleeding in the beginning of acute diforders; but not to promote an artificial

 crifis, crifis, (as has been imagined) or otherwife he would have bled on the critical days, and not in the beginning of a fever.

Another method Hippocrates took to moderate the violence of acute difeafes, particularly pleurifies, peripneumonics, and ardent fevers, was by giving cooling and emollient clyfters. In pneumonic complaints he limits the exhibition of thefe to the firft four or five days, as no evacuation was to be excited after the patient began to expectorate, left he fhould die of a fuffocation. To this general rule, however, he inftances exceptions in his own practice.——The third object was to regulate the patient's diet.

For this purpofe he made ufe of two ptiffans:—Pearl barley diffolved in ten or fifteen parts of water was called ptiffana tota, or whole ptiffan; when this was ftrained, and the thinner feparated from the thicker parts, it was called ptiffana colata, or the juice of ptiffan. His rules for giving it were thefe:—the more acute the difeafe, the more thin and watery the diet; and it ought to be thinneft about the height of the diforder. In ardent fevers he feldom gave ptiffan till after the crifis; and he directs, in one paffage, not to give it till there are figns of concoction in the urine. But, in fevers attended with more moderate fymptoms, he directs whole ptiffan to be given from the firft accefs, to fupport the patient's ftrength. Throughout the whole courfe of the fever, he directs diluting liquors to be given in the greateft abundance. To promote expectoration in pleurifies and peripneumonies, he orders the ftrained ptiffan to be given mixed with honey, and directs, when the matter begins to be expectorated, to apply warm fomentations and liniments externally, to promote a maturation of it. Sweats he confidered as a critical evacuation when they appeared on the critical days; he therefore recommended artificial means to procure them; as, warm bathing, diluting freely, and fometimes anointing the patient with oil. With a view to carry off the febrile or peccant matter, Hippocrates ordered purges at the beginning and end of acute diforders; he deferred it, however, generally till the fourth day, that he might learn more diftinctly the nature of the fever. In difeafes ftrictly inflammatory, he abftained from purging while the urine was pale and crude, a fign that the febrile matter was fixed. He was againft all evacuations during the middle ftage of acute diforders; and fays, "that as the fymptoms are moft violent about the height of a difeafe, we fhould rather affift Nature in the ftruggle, than weaken her by any evacuations at that time;"—this, however, applies to continual fevers, for he purged in a tertian on the eighth day.—With refpect to purging at the conclufion of fevers, he directs, that when they terminate terminate by resolution, or a simple concoction of the febrile matter, and thereby changed into a healthy state, no purge should be given afterward. But if there has been a critical concoction or evacuation, he directs a purge for fear of a relapse *from an imperfect crisis.*

Such was the practice of this admirable physician. He calls his art "the most excellent of all arts," and it lost not its value in the hands of such an artist.—Penetrating and indefatigable, he studied Nature closely. No symptom, no indication escaped him; and by restricting his practice to the suggestions of the complaint, he anticipated their approach, and seldom failed to give relief.

His doctrine, however, though it stood its ground for four hundred years, was found too simple for the profound speculators of after-times; and Asclepiades rejected it and set up another, which happily did not last long, but was overturned by Themison, who cured all diseases by bleeding, purging, and cold water. Theories now multiplied fast, and new systems of medicine appeared every day. At length came Galen, who seemed born for the restoration of the Hippocratic practice. In acute diseases particularly, he never failed to tread in the steps of his illustrious predecessor, whether with regard to assisting or restraining Nature; purging at the access and decline of fevers, ordering expectorating medicines after concoction, or any other dicta of so eminent an example.

After the revival of the Hippocratic practice by Galen, it stood firm for many centuries; but it was once more destined to sink under opposition. Argentarius, Pereira, and Fernelius began the attack on the Galenic mode, which gradually declined till Paracelsus and Van Helmont gave it a fatal blow. After a second eclipse of its merit it has partially revived, within the last century and a half, under the fostering hands of Sydenham and Boerhaave, who incorporated the ancient practice with their own, and who, we may safely add, owe more of their reputation to the diligent study and imitation of Hippocrates, than to any other cause.

A popular writer observes, that "the difference between a good physician and a bad one, is very great; but the difference between a good physician and *none at all*, is oftentimes very little. Hippocrates was a medical centinel; he was all eye, ear, and vigilance. Every thing relative to a complaint was minutely attended to; and with a dexterity of inference, unknown to modern practitioners, he knew what was to be done, and the precise moment for doing it.

Theories are but the butterflies of the day—they buzz for a while, and then expire. We can trace, for many centuries past, paſt, one theory overturning another, yet each in ſucceſſion promiſing itſelf immortality. Who imagined that the doctrine of phlogiſton was to be overturned ſo ſoon? —In fifty years more it may be forgotten.

But the practice of Hippocrates will endure for ever; for it is founded in truth, whoſe immutability neither time nor accident can control.

